# Socially Compliant Path Planning for Robotic Autonomous Luggage Trolley Collection at Airports

**DOI:** 10.3390/s19122759

**Published:** 2019-06-19

**Authors:** Jiankun Wang, Max Q.-H. Meng

**Affiliations:** Department of Electronic Engineering, The Chinese University of Hong Kong, Hong Kong, China; jkwang@ee.cuhk.edu.hk

**Keywords:** mobile robot navigation, path planning, autonomous robots, self-organizing map, potential field

## Abstract

This paper describes a socially compliant path planning scheme for robotic autonomous luggage trolley collection at airports. The robot is required to efficiently collect all assigned luggage trolleys in a designated area, while avoiding obstacles and not offending the pedestrians. This path planning problem is formulated in this paper as a Traveling Salesman Problem (TSP). Different from the conventional solutions to the TSP, in which the Euclidean distance between two sites is used as the metric, a high-dimensional metric including the factor of pedestrians’ feelings is applied in this work. To obtain the new metric, a novel potential function is firstly proposed to model the relationship between the robot, luggage trolleys, obstacles, and pedestrians. The Social Force Model (SFM) is utilized so that the pedestrians can bring extra influence on the potential field, different from ordinary obstacles. Directed by the attractive and repulsive force generated from the potential field, a number of paths connecting the robot and the luggage trolley, or two luggage trolleys, can be obtained. The length of the generated path is considered as the new metric. The Self-Organizing Map (SOM) satisfies the job of finding a final path to connect all luggage trolleys and the robot located in the potential field, as it can find the intrinsic connection in the high dimensional space. Therefore, while incorporating the new metric, the SOM is used to find the optimal path in which the robot can collect the assigned luggage trolleys in sequence. As a demonstration, the proposed path planning method is implemented in simulation experiments, showing an increase of efficiency and efficacy.

## 1. Introduction

At airports, a large number of luggage trolleys are used to help passengers carry their luggage to departure gates and the transit center. For example, serving a passenger traffic of 72.9 million annually, the Hong Kong International Airport has to handle around 13,000 luggage trolleys distributed in 2 terminals. The scattered trolleys within the airport have brought difficulty to the maintenance as they need to be redistributed manually to designated locations. For the purpose of controlling the cost of labor and time, it is natural to consider implementing a robotic autonomous luggage trolley collection system at airports.

This work discusses the path planning scheme for a mobile robot to collect luggage trolleys through formulating it as a Traveling Salesman Problem (TSP). Despite the dynamic and complicated environment of an airport, without loss of generality, the states of the robots, luggage trolleys, obstacles, and pedestrians are assumed to be known by the image processing of data from the Central Closed-Television (CCTV).

In the original TSP [1], the Euclidean distance of a line directly connecting two cities is usually used as the metric to construct the final solution. Easy though it may seem, direct application of that method for the current problem is not valid due to the limits listed below. Firstly, the solution is far from the real application as the path needs to be re-planned to avoid obstacles and pedestrians based on their ever-changing positions prior to the first movement of the mobile robot. With more obstacles in the planned path obtained from the initial TSP method, this need is more obvious. Secondly, the kinematic and dynamic constraints of the robot could not be taken into consideration while constructing the solution using the TSP method. Thirdly, the pedestrians might be offended as the TSP method could only calculate a feasible path without examining the potential invasion of the pedestrians’ personal space [2,3], especially in environments with massive passenger flow, like an airport.

In contrast, the potential filed method [4] and the Self-Organizing Map (SOM) [5] can overcome these shortcomings and achieve socially compliant path planning [6,7,8]. On one hand, the potential field is a natural connection between path planning and control. More specially, the total virtual force generated from the potential field is widely utilized for motion and path planning. In [4,9,10], the total force is directly used as the whole or a part of the control input to the controller of the robot, whereas in [11], it is used for the steering control. On the other hand, based on the concept of the Social Force Model (SFM) [12,13], the pedestrians’ comfort level could be considered in the path planning by applying a new potential function. In this way, socially compliant path planning can be achieved. Therefore, the method to tackle this TSP can be broken down into several steps:

1. Construction of the potential field among the robot, luggage trolleys, obstacles, and pedestrians;
2. Generation of a collision-free and easily applied path connecting the robot and the luggage trolley, or two luggage trolleys, according to the total virtual force which can satisfy the kinematic and dynamic constraints;
3. Measurement of the length of the path as a new metric to solve the SOM.

For step 3, the neurons used in the SOM correspond to the points in the potential field, and the distance between each pair of them can be calculated with the new metric. Due to the self-organizing characteristic, the SOM can efficiently find the final solution to the TSP. Using this method, a feasible path in which the robot can collect the luggage trolleys in sequence could be provided. This technique, as shall be proven hereinafter, suits well in a dynamic and complicated environment full of uncertainties like the airports, fulfilling the stability and robustness requirements [14].

**Figure 1:**
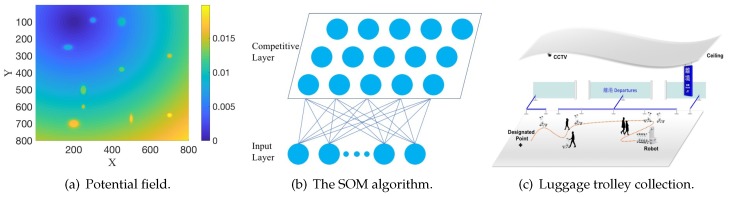
The framework of the robotic autonomous luggage trolley collection at airports.

Figure 1 illustrates the framework of the proposed method. Firstly, the luggage trolley provides an attractive force; while the obstacles and pedestrians provide a repulsive force. The robot needs to deliver all luggage trolleys to the designated point and return to its original state, so the designated point is equivalent to one luggage trolley in the potential field. The robot is considered as a point on the map, bringing no effect to the potential field. Under the influence of the attractive force and repulsive force, paths can be generated between a robot and any luggage trolley, as well as between any pairs of the luggage trolleys. As shown in Figure 1a, the positions of the luggage trolleys, obstacles, and pedestrians are considered as the input to the potential function to generate the potential field. The blue color denotes the maximum of the attractive force (the position of the luggage trolley); while the yellow color denotes the maximum of the repulsive force. The big yellow and green points denote the positions of the obstacles or pedestrians. Secondly, the lengths of the above-generated paths are considered as the new metric in the SOM. The SOM will put all luggage trolleys and the robot into a meaningful order, where any pairs of luggage trolleys, single luggage trolleys, and the robot are connected by the paths. Figure 1b illustrates the execution process of the SOM algorithm in solving the TSP. Finally, based on the result from the SOM, the final path to collect all luggage trolleys is determined. As shown in Figure 1c, with the result from the SOM, a collection sequence is obtained. In the collection sequence, the path connecting any pairs of luggage trolleys, single luggage trolley, and the robot is generated from the potential field. Finally, a feasible path (the orange line in Figure 1c) connecting all luggage trolleys and the robot is determined. When delivering all luggage trolleys to the designated point, the robot will return to its original state.

The contributions of this paper are summarized as follows:

- A socially complaint path planning scheme consisting of the potential field method and the SOM;
- A novel metric used to solve the TSP.

The rest of the paper is organized as follows. In Section 2, the related work is introduced. Then, the formulation of the robotic autonomous luggage trolley collection problem is provided in Section 3. The potential field method is elaborated in Section 4. The experimental results are presented in Section 5. Finally, conclusions are drawn and discussion of future work is presented in Section 6.

## 2. Related Work

### 2.1. TSP

In fact, the robotic autonomous luggage trolley collection problem is a variant of the TSP. The TSP has received much attention recently. The simplest model of the TSP has been extended to deal with all kinds of real applications. For example, Max TSP aims to find a possible route where the total cost is maximum. Bellmore et al. propose *m*-salesman TSP [15], where each salesman needs to visit each part of cities exactly once. In the time windows-based TSP [16], the salesman is required to visit each city at a given time. More variants of the TSP can be found in [17]. All above TSPs usually use the Euclidean distance of a line directly connecting two cities as the basic metric. In some scenarios, there also exist other TSPs with different metrics. For example, the Dubins TSP [18] is applied to the task assignment problems with kinematic constraints. In this paper, in order to adapt to the airport environment, a new metric—the length of a path generated from the potential field—is selected to solve the TSP.

### 2.2. Potential Field

Khatib et al. [4] proposed the artificial potential field in obstacle avoidance for manipulators and mobile robots. In the potential field function, the agent moves in a field of force. The position to be reached is an attractive pole for the agent, while the obstacles are repulsive surfaces for the agent. By combining the attractive force and the repulsive force in the current environment, a potential field is generated to direct the agent to arrive at the target position while avoiding the obstacles. Due to its elegant mathematical analysis and simplicity, the potential field is widely used in robot path planning and control problems.

### 2.3. SOM

In order to solve the TSP, the SOM method is implemented. The SOM method was first introduced by Kohonen [5], and later extended [19]. The SOM consists of a (usually two-dimensional) grid of nodes. A model of some real-world observation is associated with each node. Through a nonparametric and recursive regression process, the nodes are automatically organized into a meaningful order—where similar nodes are closer to each other than the dissimilar ones. By utilizing the spatial arrangement of these organizations, the SOM method can be easily applied to many real applications [20]. In the TSP domain, the SOM method also gets much attention. By implementing a competitive learning principle, the SOM method efficiently and quickly arranges the related outputs of similar inputs close to each other, where each output refers to a node in the TSP. Then, a feasible route is obtained by connecting the outputs one by one. Yamakawa et al. [21] introduced a graph structure as an input to the SOM to solve the TSP with obstacles. Zhu et al. [22] used the SOM method to solve the dynamic task assignment and path planning problems. A SOM based neural network (NN) algorithm was proposed by Zhu et al. [14] to solve the *m*-TSP. All aforementioned studies implement the SOM with the simple Euclidean distance of a line directly connecting two nodes. However, for this work the SOM is implemented to solve the TSP with the length of the path from the potential field. Although the SOM cannot compete with the best combinatorial heuristics method for the TSP, it has a significant advantage in problems where the neighborhood points [23] or the observation locations [24] need to be determined. In the current problem, there is a need to find many feasible neighborhood points in the potential field, so the SOM is applied to solve the TSP.

## 3. Problem Formulation with SOM

This section discusses the formulation of the socially complaint path planning problem with the SOM.

The potential field-based path planning for robotic autonomous luggage trolley collection is to plan an appropriate path (e.g., with the smallest cost under current metric) for the mobile robot to collect all assigned luggage trolleys in the designated area, as shown in Figure 1c. Obviously, it is a variant of the TSP. Here some slight modifications are conducted on the SOM to deal with the TSP. The details of the SOM algorithm are shown in Algorithm 1. The metric in the SOM is the length of the path generated from the potential field, which is described in detail in Section 4.

**Algorithm 1:** SOM Algorithm.

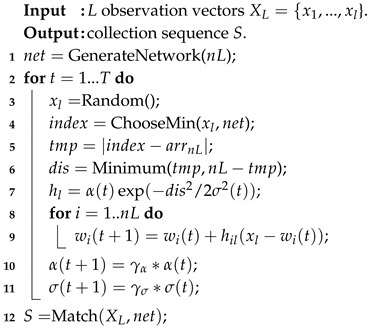



As introduced in Section 2.3, the SOM is a two-layered neural network, as shown in Figure 1b. In the input layer, a set of *L* observation vectors $X_{L}$, consisting of the coordinate information of the robot and the luggage trolley, is provided. In the competitive layer (also called the output layer), **GenerateNetwork(nL)** generates nL neurons $w_{i}\in net$ with random values, where *n* is used to balance the exploration and exploitation of the algorithm. In fact, these neurons are random points located in the potential field, and the distance between each pair of them can be calculated through the negative gradient descent method.

**Figure 2:**
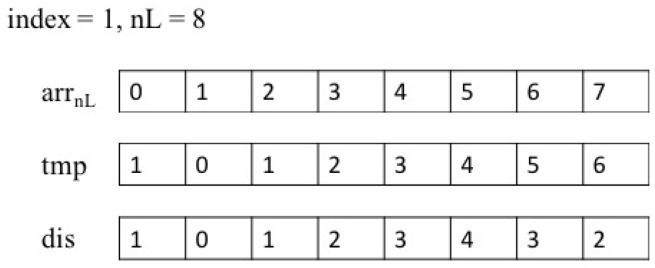
The illustration of index distance calculation.

In each iteration, firstly, **Random()** randomly chooses an observation vector $x_{l}$. Then, **ChooseMin($x_{l},net$)** calculates the distance under the potential field between $x_{l}$ and all neurons $w_{i},\in ,net$, and returns the index of the neuron (usually called the “winner”) which is the closest to $x_{l}$. In order to get a circular array of neurons, the neighborhood function $h_{l}$ is designed to make each $x_{l}$ only conscious of the neurons in front of and behind it. $arr_{nL}$ is an array to store the index of all neurons, and tmp is also an array. Figure 2 illustrates the index distance calculation. Suppose there are 8 neurons and the index of the “winner” neuron is 1, the dis value of each neuron is calculated to behave as an elastic ring. This means that all neurons are getting closer to the “winner” neuron from the front and the back. Through **Minimum(tmp,nL−tmp)**, if one neuron is closer to the “winner” neuron $w_{index}$ in the order of index (from the front and the back, as shown in Figure 2), the dis value of this neuron will be smaller. Therefore, the self-organizing map will behave as an elastic ring, getting closer to the observation vectors but trying to minimize the perimeter of itself due to the neighborhood function. $\gamma_{\alpha }$ and $\gamma_{\sigma }$ are the learning rates to ensure the convergence of the algorithm. The initial value of α(t) is large in order to obtain high exploration first, then it will decrease monotonically in the regression progress to obtain high exploitation. σ(t) is the neighborhood dispersion (the width of the neighborhood function), and it is also required to decrease monotonically for the purpose of the algorithm convergence.

After the whole regression progress is completed, the function **Match**$\left(X_{L},net\right)$ is used to find the best match in the neurons $w_{i}$ for each observation vector $x_{l}$. All neurons $w_{i}$ are arranged in a meaningful order according to their distribution in the potential field. Then, after arranging all observation vectors $x_{i}$ in terms of the order of their corresponding neurons, a feasible route connecting all $x_{l}$ is obtained.

## 4. Potential Field Method

In this section, the novel metric used in the SOM is introduced.

As is known, there are two kinds of forces in the potential field. In this paper, the attractive force comes from the position of each luggage trolley; and the repulsive force comes from the position of the obstacles and the pedestrians.

Before further discussing these two forces, the following assumptions have to be made:

- *Assumption 1.* The state (including the position ${\vec{p}}_{r}$ and the orientation ${\vec{q}}_{r}$) and velocity ${\vec{v}}_{r}$ of the robot are prior known.
- *Assumption 2.* The position ${\vec{p}}_{l}$ of each luggage trolley and the position of each obstacle ${\vec{p}}_{o}$ are prior known.
- *Assumption 3.* The position ${\vec{p}}_{p}$ and velocity ${\vec{v}}_{p}$ of each pedestrian are prior known.

As mentioned above, these assumptions are reasonable because all the above information can be easily obtained through CCTV, obstacle detection methods [25,26], and corresponding image processing techniques. In the path planning process, the next state of the robot will be calculated based on current position ${\vec{p}}_{r}$, orientation ${\vec{q}}_{r}$, and velocity ${\vec{v}}_{r}$ of the robot.

### 4.1. Attractive Potential Function

Conventional potential function takes the following form:

(1) $${\vec{U}}_{att}\left({\vec{p}}_{r}\right)=\xi \rho^{m}\left({\vec{p}}_{r},{\vec{p}}_{l}\right),$$

where ξ is the positive scaling parameter, $\rho \left({\vec{p}}_{r},{\vec{p}}_{l}\right)=\left|\right|{\vec{p}}_{r}-{\vec{p}}_{l}\left|\right|$ is the distance between the robot ${\vec{p}}_{r}$ and the luggage trolley ${\vec{p}}_{l}$, and m=1 or 2. Here, we set m=2, and the attractive potential is parabolic in shape. For better performance, the attractive potential is calculated differently as the distance between the robot and the luggage trolley changes. The attractive potential function is as follows:

(2) $${\vec{U}}_{att}\left({\vec{p}}_{r}\right)=\left\{\begin{array}{c} \xi \rho^{2}\left({\vec{p}}_{r}-{\vec{p}}_{l}\right),\;\;\;\;\;\rho \left({\vec{p}}_{r},{\vec{p}}_{l}\right)\leq \rho_{d}\\ \xi \left(\rho \left({\vec{p}}_{r},{\vec{p}}_{l}\right)\rho_{d}-\rho_{d}^{2}\right),\;\rho \left({\vec{p}}_{r},{\vec{p}}_{l}\right)>\rho_{d}. \end{array}\right.$$

The attractive force is defined by the negative gradient of the attractive potential

(3) $${\vec{F}}_{att}\left({\vec{p}}_{r}\right)=-\nabla_{p}{\vec{U}}_{att}\left({\vec{p}}_{r}\right)=\left\{\begin{array}{c} -2\xi \left({\vec{p}}_{r}-{\vec{p}}_{l}\right),\;\;\rho \left({\vec{p}}_{r},{\vec{p}}_{l}\right)\leq \rho_{d}\\ -\xi \rho_{d}\frac{{\vec{p}}_{r}-{\vec{p}}_{l}}{\rho ({\vec{p}}_{r}-{\vec{p}}_{l})},\;\rho \left({\vec{p}}_{r},{\vec{p}}_{l}\right)>\rho_{d}, \end{array}\right.$$

where $\rho_{d}$ denotes the radius of the circle centered at ${\vec{p}}_{l}$. In the original attractive potential function, when $\rho ({\vec{p}}_{r}-{\vec{p}}_{l})$ is very large, the attractive force will also become very large. This means that when the robot is far away from the luggage trolley, it is easily guided to move too close to pedestrians or obstacles. In real applications, the robot has a risk of collision with the pedestrians or obstacles when there is some error or uncertainty in path planning. Therefore, the original function is adjusted to $\xi (\rho \left({\vec{p}}_{r},{\vec{p}}_{l}\right)\rho_{d}-\rho_{d}^{2})$ to avoid this problem when $\rho \left({\vec{p}}_{r},{\vec{p}}_{l}\right)>\rho_{d}$. Finally, as the robot approaches the luggage trolley, ${\vec{F}}_{att}\left({\vec{p}}_{r}\right)$ converges to 0.

### 4.2. Repulsive Potential Function for Obstacles

Consider the airport scenario, obstacles are usually static, while passengers may stand or walk around the airport. Therefore, the repulsive force from obstacles and passengers should not be calculated in the same way. In our paper, inspired by the work in [27], the repulsive potential function is separated into two parts—one for the obstacles, and the other for the passengers.

The repulsive potential function for obstacles takes the conventional form as follows:

(4) $${\vec{U}}_{rep1}\left({\vec{p}}_{r}\right)=\left\{\begin{array}{cc} \frac{1}{2}\eta {\left(\frac{1}{\rho ({\vec{p}}_{r},{\vec{p}}_{o})}-\frac{1}{\rho_{0}}\right)}^{2}, & \rho \left({\vec{p}}_{r},{\vec{p}}_{o}\right)\leq \rho_{0}\\ 0, & \rho \left({\vec{p}}_{r},{\vec{p}}_{o}\right)>\rho_{0}, \end{array}\right.$$

where η is a positive scaling parameter, $\rho \left({\vec{p}}_{r},{\vec{p}}_{o}\right)=\left|\right|{\vec{p}}_{r}-{\vec{p}}_{o}\left|\right|$ is the distance between the robot ${\vec{p}}_{r}$ and the obstacle ${\vec{p}}_{o}$, and $\rho_{0}$ is a constant denoting the distance of influence from the obstacle.

The repulsive force is also defined by the negative gradient of the repulsive potential

(5) $${\vec{F}}_{rep1}\left({\vec{p}}_{r}\right)=-\nabla {\vec{U}}_{rep1}\left({\vec{p}}_{r}\right)=\left\{\begin{array}{cc} \eta \left(\frac{1}{\rho ({\vec{p}}_{r},{\vec{p}}_{o})}-\frac{1}{\rho_{0}}\right)\frac{1}{\rho^{2}\left({\vec{p}}_{r},{\vec{p}}_{o}\right)}\frac{{\vec{p}}_{r}-{\vec{p}}_{o}}{\rho ({\vec{p}}_{r}-{\vec{p}}_{o})}, & \rho \left({\vec{p}}_{r},{\vec{p}}_{o}\right)\leq \rho_{0}\\ 0, & \rho \left({\vec{p}}_{r},{\vec{p}}_{o}\right)>\rho_{0} \end{array}\right.$$

### 4.3. Repulsive Potential Function for Pedestrians

The two potential functions above only consider the Euclidean distance among the robot, luggage trolley, and the obstacles, but the repulsive potential function for the pedestrians takes additional factors into consideration due to the complex dynamics of the pedestrians. More importantly, attention has to be paid to the pedestrians’ feelings because the robotic autonomous luggage trolley collection system will be applied at the airport where the passenger flow is large. If the movement of the robot causes disruption to the pedestrians, then it is meaningless to design such a kind of robotic autonomous collection system.

In [12], Helbing and Molnar propose a social force model for pedestrian dynamics. The model is complicated, and is designed as follows:

(6) $${\vec{F}}_{\alpha }\left(t\right):={\vec{F}}_{\alpha }^{0}\left({\vec{v}}_{\alpha },{\vec{v}}_{\alpha }^{0}{\vec{e}}_{\alpha }\right)+\sum_{i}{\vec{F}}_{\alpha i}\left({\vec{e}}_{\alpha },{\vec{r}}_{\alpha }-{\vec{r}}_{i},t\right)+\sum_{B}{\vec{F}}_{\alpha B}\left({\vec{e}}_{\alpha },{\vec{r}}_{\alpha }-{\vec{r}}_{B}^{\alpha }\right)+\sum_{\beta }{\vec{F}}_{\alpha \beta }\left({\vec{e}}_{\alpha },{\vec{r}}_{\alpha }-{\vec{r}}_{\beta }\right),$$

where ${\vec{F}}_{\alpha }^{0}$ and ${\vec{F}}_{\alpha i}$ denote the attractive effects from a certain destination and other pedestrians, and ${\vec{F}}_{\alpha \beta }$ and ${\vec{F}}_{\alpha B}$ denote the repulsive effects from other pedestrians and obstacles.

In fact, this social force model is similar to the potential field method because they both consider using the attractive and repulsive force to construct a certain relationship in their own models. In order to take the pedestrians’ feelings into consideration, a novel potential function is proposed for the pedestrians by combining a modified social force model.

In order to make our robotic system not affect the pedestrians, the designer needs to consider the influence from the robot, which is similar to ${\vec{F}}_{\alpha i}$ and ${\vec{F}}_{\alpha \beta }$. Naturally, the path of the robot should avoid affecting the pedestrians as much as possible. Therefore, there only exists the repulsive force from the robot. In the social force model, the relative velocity is used to calculate the final total force. Inspired by it, the relative velocity between the robot and the pedestrians is used to construct the potential function. First, the relative velocity is calculated as follows:

(7) $${\vec{v}}_{re}={\vec{v}}_{r}-{\vec{v}}_{p}$$

If the maximum deceleration of the robot is ${\vec{a}}_{max}$, then the minimum distance traversed by the robot before ${\vec{v}}_{re}$ reduces to 0 is

(8) $$s=\frac{{\vec{v}}_{re}^{2}}{2{\vec{a}}_{max}}.$$

In order to control the minimum distance that can occur, a positive scaling factor $\rho_{re}>1$ is used as follows:

(9) $$\rho_{m}\left({\vec{v}}_{re}\right)=\rho_{re}\cdot \frac{{\vec{v}}_{re}^{2}}{2{\vec{a}}_{max}}.$$

Finally, the novel repulsive potential function is

(10) $${\vec{U}}_{rep2}\left({\vec{p}}_{r},{\vec{v}}_{r}\right)=\left\{\begin{array}{cc} \frac{1}{2}\eta {\left(\frac{1}{\rho \left({\vec{p}}_{r},{\vec{p}}_{p}\right)-\rho_{m}\left({\vec{v}}_{re}\right)}-\frac{1}{\rho_{0}}\right)}^{2}, & \mathrm{if}\;\rho \left({\vec{p}}_{r},{\vec{p}}_{p}\right)-\rho_{m}\left({\vec{v}}_{re}\right)\leq \rho_{0}\\ 0, & \mathrm{if}\;\rho \left({\vec{p}}_{r},{\vec{p}}_{p}\right)-\rho_{m}\left({\vec{v}}_{re}\right)>\rho_{0}. \end{array}\right.$$

Correspondingly, the repulsive force ${\vec{F}}_{rep2}\left({\vec{p}}_{r},{\vec{v}}_{r}\right)$ is defined as the negative gradient of ${\vec{U}}_{rep2}\left({\vec{p}}_{r},{\vec{v}}_{r}\right)$ in terms of both position and velocity

(11) $$\begin{array}{cc} {\vec{F}}_{rep2}\left({\vec{p}}_{r},{\vec{v}}_{r}\right) & =-\nabla {\vec{U}}_{rep2}\left({\vec{p}}_{r},{\vec{v}}_{r}\right)\\ & =-\nabla_{p}{\vec{U}}_{rep2}\left({\vec{p}}_{r},{\vec{v}}_{r}\right)-\nabla_{v}{\vec{U}}_{rep2}\left({\vec{p}}_{r},{\vec{v}}_{r}\right), \end{array}$$

where

(12) $$\begin{array}{c} \nabla_{p}{\vec{U}}_{rep2}\left({\vec{p}}_{r},{\vec{v}}_{r}\right)=\frac{\partial {\vec{F}}_{rep2}\left({\vec{p}}_{r},{\vec{v}}_{r}\right)}{\partial \vec{p}}\\ \nabla_{v}{\vec{U}}_{rep2}\left({\vec{p}}_{r},{\vec{v}}_{r}\right)=\frac{\partial {\vec{F}}_{rep2}\left({\vec{p}}_{r},{\vec{v}}_{r}\right)}{\partial \vec{v}}. \end{array}$$

The subscripts $\vec{p}$ and $\vec{v}$ in Equation (12) denote the gradient in terms of the position and the velocity, respectively.

With the attractive force ${\vec{F}}_{att}\left({\vec{p}}_{r}\right)$ and the repulsive force ${\vec{F}}_{rep1}\left({\vec{p}}_{r}\right)$, ${\vec{F}}_{rep2}\left({\vec{p}}_{r},{\vec{v}}_{r}\right)$ mentioned above, the total force applied to the robot is

(13) $$\vec{F}={\vec{F}}_{att}\left({\vec{p}}_{r}\right)+\sum_{i=1}^{n_{o}}{\vec{F}}_{rep1}\left({\vec{p}}_{r}\right)+\sum_{j=1}^{n_{p}}{\vec{F}}_{rep2}\left({\vec{p}}_{r},{\vec{v}}_{r}\right),$$

where $n_{o}$ and $n_{p}$ denote the number of obstacles and pedestrians in the current designated area, respectively. It is noted that when calculating a path for the robot, only one luggage trolley that is located at the target position will be considered. Otherwise, it will bring a lot of local minimum points in the potential field.

### 4.4. Problem of Local Minimum

As we all know, the main challenge in the potential field method is the problem of the local minimum. Even if we only consider the luggage trolley located at the target position during the calculation of the path, the problem of local minimum still exists. In this paper, we use a simple method to avoid the local minimum problem. For example, when the robot gets stuck in one certain point but has not arrived at the destination, the search range will be set around this point. In this search range, the robot will try to find one point with lower potential, then it will move towards this point to escape from the local minimum point. The search range can be continuously enlarged until the robot escapes from the local minimum point.

## 5. Results of the Simulation Experiment

The experiments in the simulation environments demonstrated the efficacy and the efficiency of the proposed algorithm by comparing it to some conventional methods. Windows 10 on an Intel i5-4590 with 8GB RAM was used as the experimental platform. The parameter settings were as follows. In the SOM algorithm, α(0) = 0.80, σ(0) = 1.00, $\gamma_{\alpha }$ = 0.07, and $\gamma_{\sigma }$ = 0.99. In the potential field method, ξ = 2.00 and η = 2.00. In the conventional ant colony optimization algorithm, the number of ants *K* = 50, the update rate of pheromone information $A_{\alpha }$ = 1.00, the update rate of heuristic information $A_{\beta }$ = 2.00, and the evaporation parameter ρ = 0.20.

**Figure 3:**
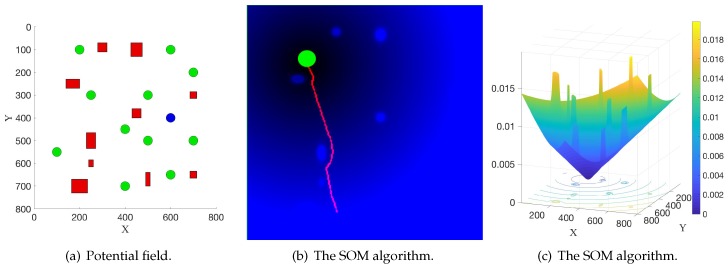
Socially compliant path planning for robotic autonomous luggage trolley collection.

Figure 3 illustrates the socially compliant path planning for robotic autonomous luggage trolley collection in a scenario with some obstacles and luggage trolleys. As shown in Figure 3a, the red rectangles, the green circles, and the blue circle denote the obstacles of different sizes, the luggage trolleys, and the robot, respectively. Figure 3b illustrates the potential field generated by one luggage trolley and all other obstacles, where the black color means a low potential; and the blue color means a high potential. The red line denotes a feasible path from one certain point to the position (green circle) of the luggage trolley, which was generated by the negative gradient descent method. It is noted that at each iteration, only one luggage trolley located at the target position was taken into consideration to calculate the potential field. Otherwise, there exists more than one local minimum point in the potential field, and thus the negative gradient descent method in the potential field does not work. Figure 3c is the same potential field from a different view. The yellow color means a high potential; while the blue color means a low potential. Similarly, in the SOM, the length of other paths can be obtained in the same way. Finally, through the self-organizing ability of the SOM, one appropriate path to collect all luggage trolleys could be generated.

**Figure 4:**
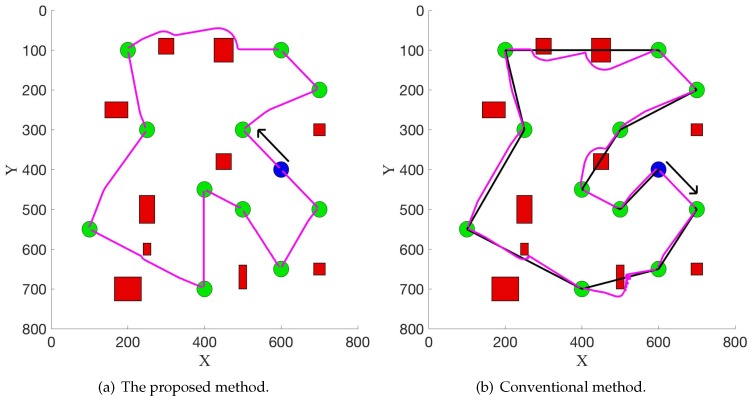
Experimental results in the first scenario.

Figure 4 illustrates the experimental results in the first scenario. The direction of the black arrow denotes the direction of the collection sequence. Figure 4a shows the result from the proposed method, and the magenta line denotes the final generated path. The conventional solution (Ant Colony Optimization [28]) to the TSP is shown in Figure 4b, where the Euclidean distance of a line directly connecting two nodes is used as the metric. This solution, denoted as the black line, needs additional path planning to satisfy the requirements in a real application, and the final path (generated from the potential field method) is denoted as the magenta line. Compared with the solution in Figure 4a, it is not optimal or appropriate, as the conventional solution does not consider environmental information, such as the obstacles. Therefore, in a real application, the robot needs to do additional path planning with such a solution to avoid the obstacles. This probably results in a bad final path. For example, the length of the final path is very long. However, the proposed method takes environmental information into consideration and directly produces a feasible path for the robot without requiring extra path planning in the execution process. In addition, the elastic band method [29], which connects the path planning and control, can also be naturally combined when updating the path in real time in the robot navigation process. Therefore, the robot can be controlled directly to move towards the target position. This demonstrates the efficiency of the proposed method. In fact, as shown in Figure 4, the length of the final path from the proposed method and the conventional method are 2662 and 2800, respectively. This means that this new method can help reduce 5% in the length of the whole path. This experiment is repeated 30 times, and the obstacles are randomly placed in each experiment. The average improvement is 5.2%. When the number of luggage trolleys increases to 20 and 40, the average improvements are 9.6% and 22.1%, respectively. This indicates that the length of the final path will be reduced more as the robot needs to collect more luggage trolleys, which could demonstrate the efficacy of this proposed method.

It is noted that this new method solves an asymmetric TSP because the length of the path from point A to point B does not equal the length of the path from point B to point A in the potential field. Conversely, the conventional method solves a symmetric TSP and then uses the potential field method to calculate the final path. Therefore, the robot can start moving from two opposite directions, one of which is shown in Figure 4b. When starting from the opposite direction, the length of the path is 2860. This new method can also reduce the length of the path by 7% compared with the conventional method.

**Figure 5:**
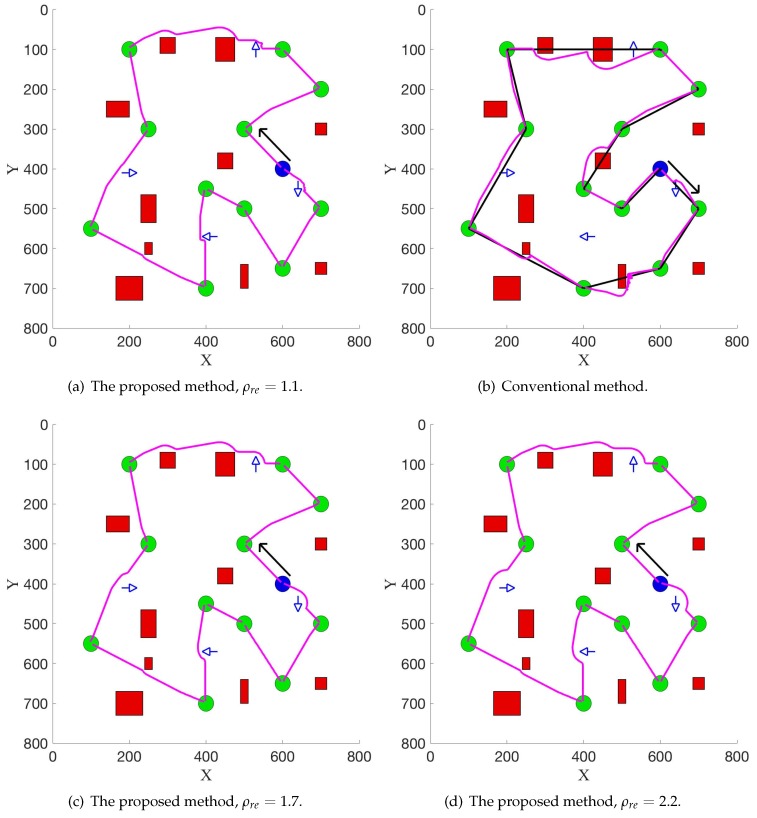
Experimental results in the second scenario.

Figure 5 illustrates the experimental results in the second scenario, where moving pedestrians are added. Figure 5a,b show the results from the proposed method and the conventional method with the same scaling parameter $\rho_{re}$, while Figure 5a,c,d show the results from the proposed method with different scaling parameter $\rho_{re}$. The direction of the blue arrow denotes the moving direction of the pedestrians. The direction of the black arrow denotes the direction of the collection sequence. In these experiments, the current velocity of the robot ${\vec{v}}_{r}$ is 5 pixel/s and the velocity of each pedestrian ${\vec{v}}_{p}$ is 9 pixel/s.

Firstly, the results in this simulation experiment demonstrate that by using the new potential function Equation (13), the robot successfully avoided the pedestrians on the way to the target position. In Figure 5a,b, the same scaling parameter $\rho_{re}=1.1$ is used and the length of the path is 2698 and 2900, which shows that the proposed method can reduce 7% in the length of the path compared with the conventional method. Secondly, one can see that in a real application, people can adjust the value of $\rho_{re}$ to satisfy different requirements. In Figure 5a,c,d, with a different value of the scaling parameter $\rho_{re}$, the path changes and the robot can be away from the pedestrians in different distances.

To further demonstrate the efficacy and efficiency of the proposed algorithm, a real-time path planning experiment in a simulated environment was implemented. This simulation experiment demonstrates how the robot collects one luggage trolley according to the collection sequence.

**Figure 6:**
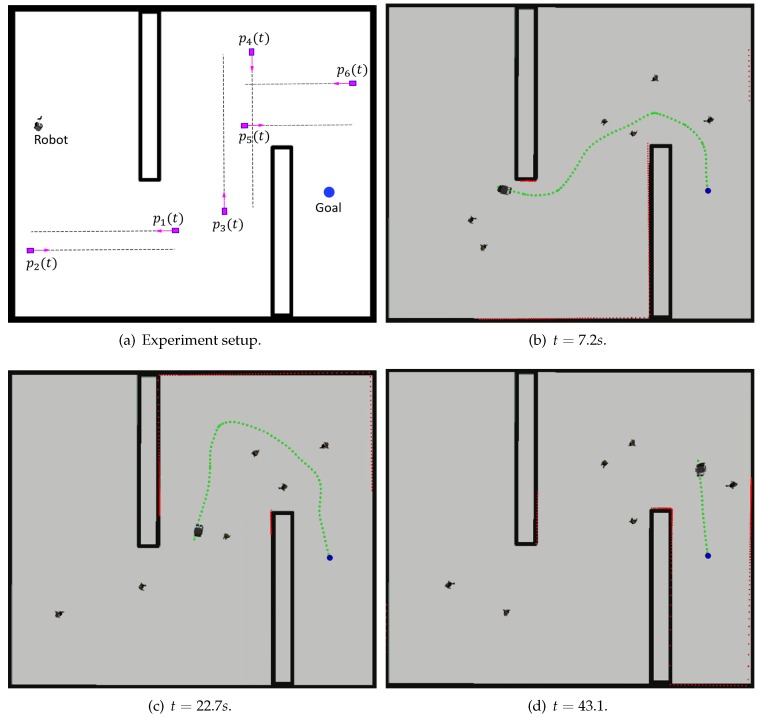
Real-time path planning experiment.

Figure 6a illustrates the experimental setup. In this simulation experiment, a mobile robot is required to track the planned path to arrive at the position of the luggage trolley. The black rectangle denotes the static obstacle while the magenta box denotes the pedestrian moving forward and backward along the dashed line at a random speed. The robot needs to avoid the static obstacles and the moving pedestrians simultaneously. Figure 6b–d illustrate the robot execution process. Firstly, as shown in Figure 6b, a feasible path (denoted as the green dashed line) is provided by the potential field method and the robot begins to move. In the execution process, the path is changed due to the movement of the pedestrians. As shown in Figure 6c, the path changes a lot so that the robot does not offend the moving pedestrians. This shows that the proposed novel metric works well, and the pedestrian is not considered as an ordinary obstacle. Finally, in Figure 6d, the robot successfully evaded all static obstacles and moving pedestrians, and approached the goal position. Therefore, the result of this simulation experiment shows that the proposed method can work well in the robot execution process.

Actually, the number of obstacles and the velocity of passengers affect the solution. Through a number of simulation experiments (the experiment environment is shown in Figure 6), as the number of passengers or the velocity of passengers increases, the success rate of the real-time path planning method decreases. Although the proposed method can provide a feasible solution, the robot fails to track the planned path. The reason is that the robot does not have sufficient reaction time before the collision in such a complex environment. In order to solve this problem, motion planning and control techniques are required. Two methods will be considered in future work. Firstly, the clearance between the robot and the obstacles should increase to provide the robot with more reaction time. This means that the weight of the repulsive potential function in the proposed method needs to be increased. Secondly, more efficient control methods should be implemented in the real-time planning process.

## 6. Conclusions and Future Work

This work has proposed a socially compliant path planning scheme, and implemented the SOM method to achieve the robotic autonomous luggage trolley collection at airports. The luggage trolley collection problem is formulated as a TSP with the SOM. The length of the path generated from the potential field is used as the new metric in the SOM. For constructing the potential field, a novel potential function is applied by taking pedestrian information into consideration. Finally, the SOM provides a final feasible path for the robot to collect all luggage trolleys. The experimental results demonstrate that the proposed method is superior to the conventional method due to its efficiency and efficacy.

However, there are also some limitations to this method. Firstly, the local minimum problem is not completely solved. The authors have considered combining the Rapidly-exploring Random Tree (RRT) [30,31] to solve this problem and use a potential field-based sampling heuristic to guide the global path planning [32]. Secondly, efforts will be paid to develop the multi-robot autonomous luggage trolley collection system because an appropriate number of robots are required at the airport. Finally, our research group is planning to further improve the performance of this method and implement practical experiments at a real airport environment, like the Hong Kong International Airport, in the near future.

## Abbreviations

The following abbreviations are used in this manuscript:

TSP	Traveling Salesman Problem
SOM	Self-Organizing Map
SFM	Social Force Model
RRT	Rapidly-exploring Random Tree
